# Dishevelled Binds the Discs Large ‘Hook’ Domain to Activate GukHolder-Dependent Spindle Positioning in *Drosophila*

**DOI:** 10.1371/journal.pone.0114235

**Published:** 2014-12-02

**Authors:** Joshua D. Garcia, Evan B. Dewey, Christopher A. Johnston

**Affiliations:** Department of Biology, University of New Mexico, Albuquerque, New Mexico, United States of America; Institut Pasteur, France

## Abstract

Communication between cortical cell polarity cues and the mitotic spindle ensures proper orientation of cell divisions within complex tissues. Defects in mitotic spindle positioning have been linked to various developmental disorders and have recently emerged as a potential contributor to tumorigenesis. Despite the importance of this process to human health, the molecular mechanisms that regulate spindle orientation are not fully understood. Moreover, it remains unclear how diverse cortical polarity complexes might cooperate to influence spindle positioning. We and others have demonstrated spindle orientation roles for Dishevelled (Dsh), a key regulator of planar cell polarity, and Discs large (Dlg), a conserved apico-basal cell polarity regulator, effects which were previously thought to operate within distinct molecular pathways. Here we identify a novel direct interaction between the Dsh-PDZ domain and the alternatively spliced “I3-insert” of the Dlg-Hook domain, thus establishing a potential convergent Dsh/Dlg pathway. Furthermore, we identify a Dlg sequence motif necessary for the Dsh interaction that shares homology to the site of Dsh binding in the Frizzled receptor. Expression of Dsh enhanced Dlg-mediated spindle positioning similar to deletion of the Hook domain. This Dsh-mediated activation was dependent on the Dlg-binding partner, GukHolder (GukH). These results suggest that Dsh binding may regulate core interdomain conformational dynamics previously described for Dlg. Together, our results identify Dlg as an effector of Dsh signaling and demonstrate a Dsh-mediated mechanism for the activation of Dlg/GukH-dependent spindle positioning. Cooperation between these two evolutionarily-conserved cell polarity pathways could have important implications to both the development and maintenance of tissue homeostasis in animals.

## Introduction

Non-random alignment of the mitotic spindle serves at least two principal functions within multicellular animals: (1) coupling spindle orientation to an axis of cortical cell polarity ensures proper segregation of cell fate determinants important for cellular differentiation, for example in asymmetric stem cell divisions, and (2) spindle positioning with respect to a tissue axis balances cell divisions that lead to tissue expansion versus stratification, for example in epithelial cells [1]. These non-mutually exclusive roles of spindle orientation are fundamental to proper development and homeostasis of complex animal tissues [2]. Defects in spindle orientation have been linked to numerous human diseases, including lissencephaly, polycystic kidney disease, and cancer [3]–[5]. However, our understanding of the molecular mechanisms linking cell polarity cues to spindle microtubules remains incomplete [6]. Although much focus has been aimed at defining linear pathways downstream of a given polarity cue, whether distinct cortical complexes can interact to cooperatively influence spindle orientation has not been extensively investigated.

Previous studies from our laboratory and others have demonstrated spindle orientation roles for two evolutionarily-conserved cell polarity proteins, Dishevelled (Dsh) and Discs large (Dlg) [7]-[14]. Dlg acts downstream of the conserved Partner of Inscuteable (Pins) protein: upon Aurora-mediated phosphorylation, phospho-Pins directly binds Dlg to initiate a microtubule capture pathway dependent on the plus-end kinesin motor, Khc-73 [9], [11], [15]. Although the Pins/Dlg complex is sufficient to induce a partial spindle alignment, robust orientation requires Pins engagement of Mushroom body defect (Mud); Pins/Mud induces spindle force generation through cytoplasmic Dynein, a minus-end microtubule motor [16]–[18]. Although additional regulatory inputs have been identified [6], this Pins/Dlg/Mud complex has been considered a distinct, albeit ubiquitous spindle orientation complex controlling oriented divisions of neural stem cells, epithelial cells, neural progenitor cells, and others [19].

**Figure 1:**
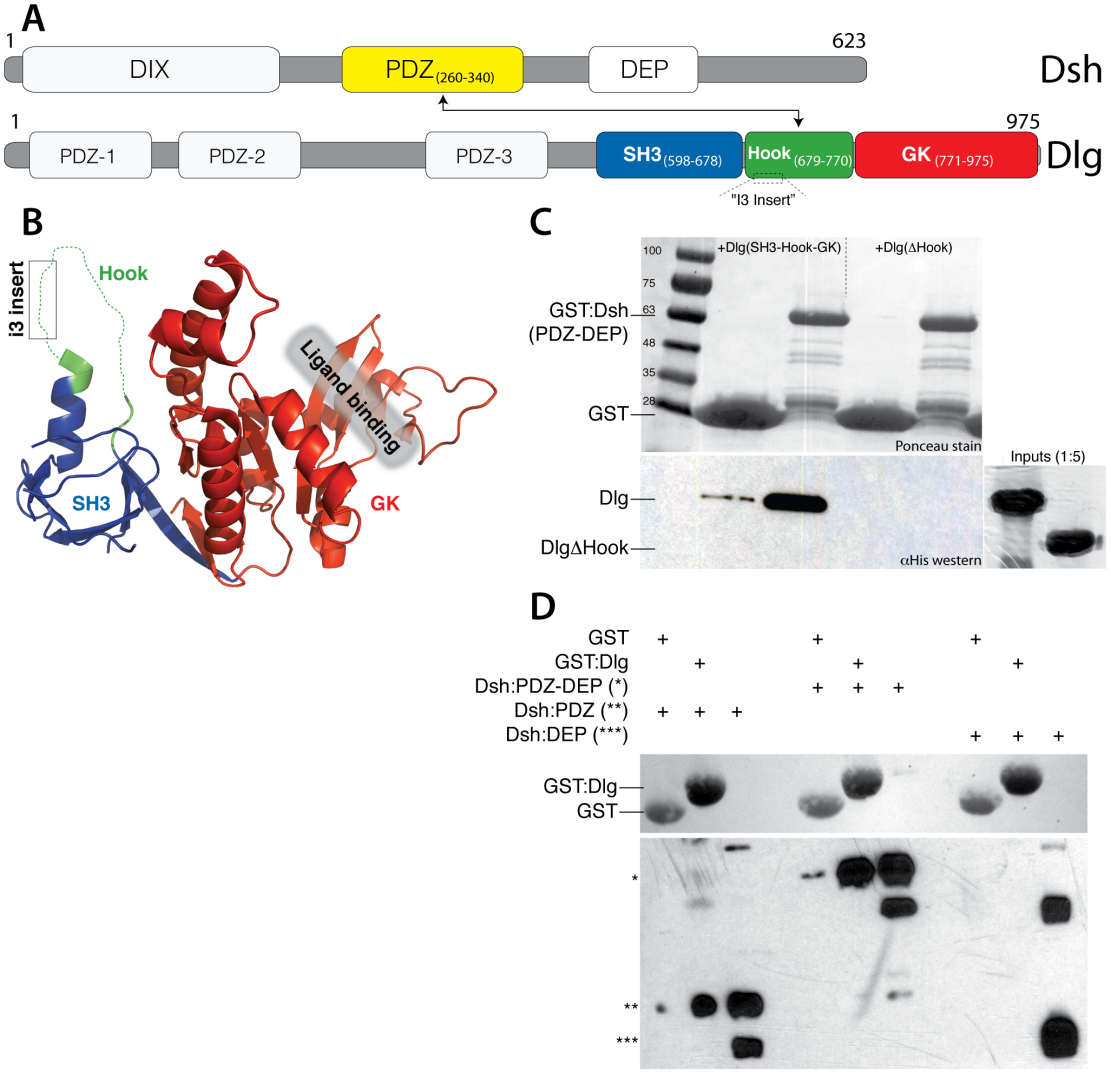
Dsh directly binds the ‘Hook’ domain of Dlg. (**A**) Domain architectures of Dsh and Dlg are shown. Dsh (*top*) consists of an N-terminal DIX (Dishevelled and Axin) domain, a central PDZ (*yellow*; Postsynaptic density-95, Discs large, and ZO-1) domain, and a C-terminal DEP (Dishevelled, Egl-10, and Pleckstrin) domain. Dlg (*bottom*) consists of tandem N-terminal PDZ domains followed by a C-terminal array of PDZ, SH3 (*blue*; Src homology-3), and GK (*red*; Guanylate Kinase) domains. The sequence connecting the SH3 and GK domains has been termed the ‘Hook’ domain (*green*), which varies in length among species (∼90 amino acids in *Drosophila*) and undergoes alternative splicing, yielding the specific ‘I3-insert’-containing isoform investigated herein. (**B**) Structural representation of the SH3-Hook-GK cassette from *Drosophila* Dlg demonstrates the close association of the SH3 and GK domains. The Hook domain is mostly absent, likely due to conformational flexibility within protein crystals. The SH3-Hook domains act as repressors of GK domain-mediated protein interactions through a poorly understood allosteric mechanism. Image rendered from PDB id: 3TVT with bound ligand removed for clarity. (**C**) GST pulldown experiments demonstrate a Hook-dependent direct interaction between Dsh and Dlg. GST alone (control) or fused to the PDZ-DEP domains of Dsh (GST:Dsh) were coupled to glutathione agarose and subsequently incubated with soluble 6x-His-tagged Dlg proteins spanning the entire SH3-Hook-GK domains (Dlg) or an SH3-GK tandem with the Hook domain removed (DlgΔHook). Samples were resolved by SDS-PAGE and analyzed by Ponceau red staining (*top*; GST proteins) or α-His western blots (*bottom*; Dlg proteins). Purified Dlg proteins input to respective reactions are shown to the *right*. (**D**) Dlg binding occurs exclusively through the PDZ domain of Dsh. GST or GST:Dlg-Hook were incubated in the presence of various Dsh domains; *left lanes* – PDZ alone (indicated by *), *middle lanes* – both PDZ and DEP domains together (indicated by **), *right lanes* – DEP domain alone (indicated by ***). Dlg-Hook-specific interactions were detected for PDZ and PDZ-DEP constructs but not for the isolated DEP domain. The top image shows the Ponceau stained membrane depicting comparable input of GST constructs across all conditions; the bottom image is the α-His western blot used to detect bound His-tagged Dsh proteins (an ‘input’ lane of each is shown for reference).

Dlg belongs to a superfamily of multidomain scaffold proteins known as Membrane Associated Guanylate Kinases (MAGUKs), a name derived from their unique C-terminal Guanylate Kinase (GK) domain [20] (Fig. 1a). The GK domain functions as a phosphoserine binding domain and is sufficient for Dlg-dependent spindle orientation [21]–[23]. Interestingly, the presence of the SH3 and Hook domains selectively repress GK domain interactions, whereas the third PDZ domain appears to relieve this autoinhibition [24]. This complex network of interdomain dynamics, thought to be controlled through a ‘supertertiary’ MAGUK structure [25], [26], regulates the spindle orientation capacity of Dlg, with the Hook domain appearing to contribute most of the GK domain inhibition [27]. Although mutational analyses have provided some biochemical insight into this process, the molecular basis for alleviating Hook-mediated repression of spindle orientation in a cellular context remains unknown.

Dsh functions downstream of the Frizzled (Fz) receptor and regulates spindle orientation through ‘noncanonical’ Wnt signaling [10], [14], [28]. Noncanonical Wnt signaling through Dsh is critical to embryonic development and maintenance of planar polarized tissue organization, and may also cooperate with convergent extension movements during morphogenesis [29]. Like Pins, Dsh associates with the spindle force-generating Mud/Dynein complex [14]. However, the Dlg/Khc-73 complex does not appear to be required downstream of Dsh; rather, Rho-mediated cortical actin polymerization provides an asymmetric spindle capture site to initiate spindle alignment [30]. Despite the apparent lack of Dlg requirement downstream of Dsh, expression of these two cell polarity cues overlap in epithelial cells of diverse tissues. In *Drosophila* sensory organ precursors, epithelial cells specialized in the formation of wing bristles, coordinated signaling between Fz/Dsh and Pins/Dlg pathways controls apico-basal polarity, spindle orientation, and cell fate specification. Specifically, Dlg fails to accumulate along the anterior cell cortex in Fz mutants, resulting in improper segregation of the cell fate determinant Numb [9], [31]. Whether direct pathway crosstalk underlies this signaling coordination has not been addressed, however.

We report here that Dsh and Dlg directly interact through their respective PDZ and Hook domains. An alternatively spliced “I3-insert” sequence within the Dlg-Hook domain is both necessary and sufficient for Dsh-PDZ binding. Furthermore, mutagenesis studies identified a K-K-x-x-x-F motif within the I3-insert required for Dsh binding. This motif resembles a putative consensus sequence for Dsh-PDZ binding first identified in the Fz receptor C-terminus [32], suggesting diverse Dsh binding partners may have acquired this short, linear sequence as an internal Dsh-PDZ ligand. Using an ‘induced polarity’ assay in *Drosophila* S2 cells, we found that Dsh expression enhances Dlg-mediated spindle orientation. This activation was equivalent to that seen following deletion of the Dlg-Hook domain, suggesting that Dsh binding provides an *in trans* mechanism for relieving the Hook-mediated autoinhibitory Dlg conformation. Finally, we report that this Dsh/Dlg-mediated spindle orientation activity is dependent on GukHolder (GukH), a Dlg-GK domain binding partner thought to associate with the actin cytoskeleton. Our results establish a novel interaction between Dsh and Dlg, two essential, evolutionarily conserved regulators of cell polarity and spindle positioning, and thus have implications to a wide range of developmental processes.

## Materials and Methods

### Molecular cloning

*Drosophila* Dlg constructs were cloned from the PG isoform and correspond to the following amino acid numbers: SH3-Hook-GK (598–975), ΔHook (598–678 + 771–975), and Hook (679–770), along with truncated sequences denoted in the text and figures. cDNA encoding these constructs were cloned into the pGEX or pMT:Ed:GFP vectors using BamHI and XhoI restriction sites. QuikChange mutagenesis (Stratagene) was used to create premature stop codons or missense amino acid mutations. Full length *Drosophila* Dsh sequence was cloned into the pMT:myc vector using BamHI and SalI restriction sites. The PDZ domain of *Danio rerio* Dvl3 (amino acids 217–352) was cloned into the pBH vector for production of 6x-His fused protein in *E. coli* [21].

### Protein purification

GST- and His-tagged proteins were expressed and purified from One-Shot BL21(DE3) *E. coli* cells (Invitrogen). GST-Hook fusions were grown to an OD_600_ ∼0.8, followed by an induction with 1 mM Isopropyl β-D-1-thiogalactopyranoside (IPTG) at 37°C for 3–4 hr. Cells were harvested and resuspended in PBS prior to sonication-mediated lysate preparation. We found that the Dsh-PDZ domain from *Drosophila* was completely recalcitrant to bacterial purification. We therefore used the *Danio rerio* Dvl3-PDZ domain, which produced small but usable yields (∼0.5 mg/L) suitable for *in vitro* experiments. His:Dvl3-PDZ was grown at 37°C to an OD_600_ ∼0.4, at which time the temperature was lowered to 18°C. Cells were allowed to reach an OD_600_ ∼0.7 prior to addition of 0.2 mM IPTG, followed by an overnight (∼16 hr) induction. Cells were harvested and resuspended in N1 buffer [50 mM Tris (pH 8), 300 mM NaCl, and 10 mM imidazole]. Following extensive sonication, lysates were incubated with ∼2 mL of Ni-Sepharose 6 Fast Flow resin (GE Healthcare) for 3 hours at 4°C with constant rocking. Samples were applied to gravity-flow columns, washed extensively [20 mM Tris (pH 8), 150 mM NaCl, and 30 mM imidazole], and eluted in N2 buffer [20 mM Tris (pH 8), 150 mM NaCl, and 300 mM imidazole]. Eluted protein was applied to a HighLoad 16/600 Superdex 200 size-exclusion column (GE Healthcare). Final purified fractions were concentrated, flash frozen in liquid nitrogen, and stored in [20 mM Tris (pH 8), 200 mM NaCl, and 2 mM dithiothreitol] at −80°C.

### GST pulldown assays

For non-quantitative *in trans* GST ‘pulldowns’, *E. coli* cell lysates (200–500 µL) expressing the GST fusion protein of interest were incubated with 100 µL of 50∶50 glutathione agarose bead slurry (Pierce) for 1 hr at room temperature with constant rocking. Beads were gently centrifuged and washed three times with wash buffer [20 mM Tris (pH 8), 150 mM NaCl, 2 mM dithiothreitol, and 1% Triton X-100]. 6x-His:Dvl3-PDZ protein (30 µg) was then incubated with bound beads for 2–3 hr at 4°C. Reaction mixtures were then washed three times with wash buffer to remove unbound or nonspecifically-associated proteins. Bound proteins were eluted from the beads by the addition of 100 µL SDS loading buffer followed by 3–5 min boiling. Samples were resolved using SDS-PAGE, transferred to nitrocellulose, and analyzed by either direct Ponceau red staining or Western blot processing using a mouse monoclonal anti-His antibody (1∶1000, Pierce).

### S2 ‘induced polarity’ assays and imaging

S2 cells were cultured at 25°C in Schneider Insect Media (Sigma) supplemented with 10% fetal bovine serum according to standard protocols [33]. For spindle orientation assays, cells were seeded in 6-well culture dishes at ∼1–3×10^6^ cells per well and transfected with 0.4–1 µg total DNA using the Effectene nonliposomal reagent (QIAGEN, Germantown, MD). Gene expression was induced with 500 µM CuSO_4_ for 24–48 hr. To reconstitute Echinoid (Ed)-mediated cell polarity, cell clustering was induced by rotation of resuspended cells at ∼175 RPM for 1–3 hr.

For RNAi treatment, primers that amplify ∼100–400 base pairs were designed using SnapDragon RNAi (http://www.flyrnai.org/snapdragon) with T7 promoter tags (TAATACGACTCACTATAGGG): Fwd-ATTGAGGATTTGCGGAAGTG/Rev- AGGCAATCTGCTTGGTCTGT (initial target); Fwd-GTTATTTTGAAACCGGCGAA/Rev- ACTGGTCGGACAACTTCTGG (‘Alt #1’ target); Fwd-TTACACGGAGGCTGGTAAGG/Rev-CTGGACAGAACCTCACCGTT (‘Alt #2’ target); Fwd-TTCGACTTGAAGCGGAACTT/Rev-GCTTCTGACTCGTTTCCGTC (‘Alt #3’ target). PCR-amplified sequences were transcribed with the Megascript T7 kit (Ambion, Austin, TX). S2 cells were seeded at 1×10^6^ cells per well in 1 ml of serum-free Schneider media and incubated with ∼1 µg of GukH RNAi. After 1 hr, 2 ml of serum-containing media was added and cells were incubated for an additional 3 days.

For immunostaining, S2 cells on glass coverslips were fixed for 20 min in 4% paraformaldehyde. Cells were washed extensively (PBS +0.1% Triton X-100) and blocked (PBS +0.1% Triton X-100 +1% BSA) for 1 hr at room temperature. Primary antibodies were incubated overnight at 4°C, followed by secondary antibody staining for 2 hr at room temperature. Cells were extensively washed between antibody treatments, and mounted using Vectashield Hardset solution. Cells were imaged on either a Zeiss 780 confocal or a Nikon Eclipse Ti microscope. Antibodies and dilutions were as follows: rat tubulin, 1∶1000 (Abcam); mouse c-Myc, 1∶1000 (Santa Cruz).

## Results and Discussion

### Dsh directly binds the Dlg-Hook domain

Our previous studies characterizing Dsh-mediated spindle orientation found that neither Dlg nor Khc-73 were required downstream effectors. Rather, RNAi against Dlg resulted in a modest improvement in Dsh function; a similar effect was seen following removal of the Dsh-PDZ domain [14]. Here we investigate the mechanism underlying these unexpected results. We hypothesized that Dsh and Dlg may participate in a convergent pathway, and thus assayed for a potential direct interaction between these key polarity proteins. Both Dsh and Dlg consist of several modular protein domains, affording a multitude of protein-protein interactions and allowing the scaffolding of diverse signaling complexes [20], [29] (Fig. 1a). The C-terminal region of Dlg contains a conserved cassette of SH3, Hook, and GK domains that typify the MAGUK family of proteins. Rather than operating as independent domains *per se*, the SH3-Hook-GK functions as a ‘supertertiary’ core unit with the GK domain providing several protein interactions [25], [26] (Fig. 1b). Canonical and noncanonical Wnt signals are transmitted through the N-terminal DIX and C-terminal DEP domains of Dsh, respectively. Dsh also contains a central PDZ domain that has been shown to associate with numerous proteins [29] (Fig. 1a). Using *in vitro* pulldown assays with purified proteins, we initially found that a fragment of Dsh spanning the PDZ-DEP domains was capable of binding the SH3-Hook-GK cassette of Dlg (Fig. 1c). Interestingly, when the Dlg-Hook domain was deleted (ΔHook) the Dsh interaction was completely abolished. We further investigated the nature of this interaction by testing each Dsh domain in isolation for binding to the Dlg-Hook region. The Dsh-PDZ domain bound Dlg similarly to the PDZ-DEP tandem, whereas the Dsh-DEP domain was void of any interaction (Fig. 1d). We conclude that the polarity proteins Dsh and Dlg bind directly *in vitro* and that the Dsh-PDZ and Dlg-Hook domains are responsible for the interaction.

### Dsh binding occurs within the Dlg I3-insert and requires a conserved PDZ binding motif

**Figure 2:**
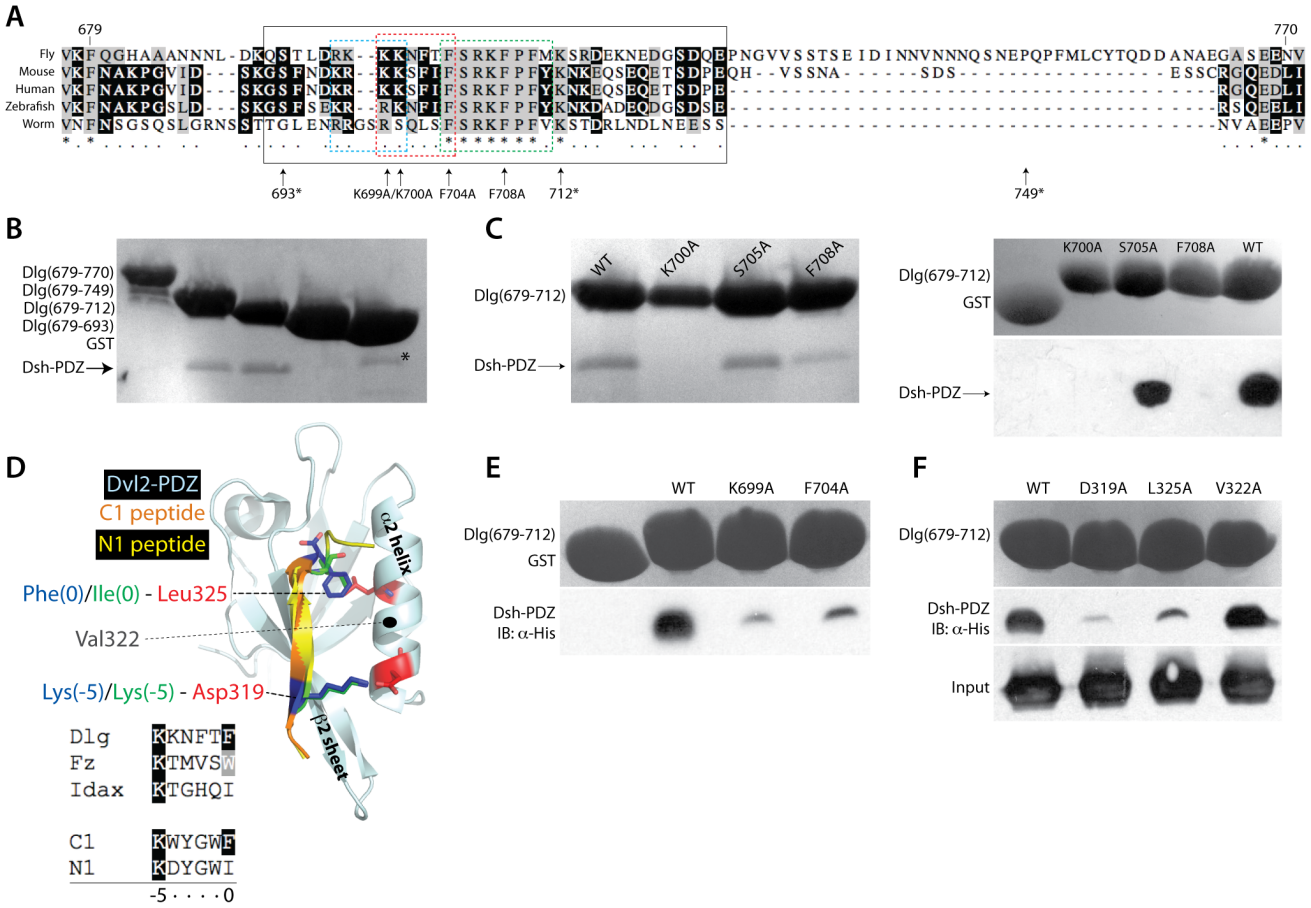
The Dsh PDZ domain binds a conserved, internal K-K-x-x-x-φ ligand motif within the Dlg I3-insert. (**A**) Multiple sequence alignment of Dlg-Hook domains reveals considerable sequence conservation across metazoan evolution. The *Drosophila* (‘Fly’) Hook domain contains a C-terminal extended sequence (amino acids 726–764) not seen in most other species. The splice isoform of Dlg studied herein includes the I3-insert sequence (*solid black box*), which contains two segments of high sequence homology across species: a polybasic region (*dashed cyan box*) followed by stretch containing several hydrophobic amino acids (*dashed green box*). The highly conserved K-K-x-x-x-φ motif identified herein is also indicated (*dashed red box*). Amino acid mutations are indicated below with numberings corresponding to fly sequence (* denotes residues chosen for ‘STOP codon’ mutations as Hook domain truncations). (**B**) The Dlg Hook domain was fused to GST and iteratively truncated from the C-terminus using site-directed mutagenesis of selected amino acids to termination codons. Immobilized GST-Dlg proteins were incubated with the purified Dsh-PDZ domain, and resolved reactions were analyzed by Ponceau red staining. The bound Dsh-PDZ protein band is indicated by an arrow; the asterisk (*) indicates a nonspecific impurity sometimes seen in the GST alone control preparation, which resolves at a slightly higher molecular weight than Dsh-PDZ. Truncation up to amino acid 712 does not affect Dsh-PDZ binding, although further Hook truncation (amino acid 693) results in a complete loss of Dsh/Dlg interaction. (**C**) Alanine mutation of conserved residues within the I3-insert were analyzed for Dsh-PDZ binding. *Left panel*: Dlg-Hook domain residues 679-712 fused to GST (either wild-type or indicated mutant) were incubated with Dsh-PDZ and bound proteins were detected using Ponceau red staining of a membrane containing transferred proteins. The K700A mutant failed to bind Dsh, whereas the S705A mutant retained full binding capacity. The F708A mutant also showed significantly reduced binding. *Right panel*: Identical experiment but with bound Dsh-PDZ detected using an α-His western blot. Results are consistent with Ponceau red analysis. (**D**) Structural representation of the human Dvl2-PDZ domain (*light cyan*) bound to two optimized phage-display peptides (*orange* and *yellow*) demonstrates the molecular basis of ligand interaction (PDB ids: 3CBX and 3CBY). Both peptides contain N-terminal lysine [Lys(-5)] and C-terminal hydrophobic [Phe(0) or Ile(0)] residues (*green* and *blue*) that make contact with conserved PDZ domain residues (*red*) within the canonical ligand binding groove. Below is shown a multiple sequence alignment of the peptides (‘C1’ and ‘N1’), together with three natural Dsh-PDZ binding partners (Fz, Idax, and Dlg), demonstrating the conservation of the (0; hydrophobic) and (-5; lysine) positions. (**E**) Alanine mutation of Dlg K699 and F704 within the **K**-K-x-x-x-**φ** motif reduce binding to Dsh-PDZ. Resolved proteins were analyzed by α-His western blot analysis. (**F**) Alanine mutation of Dsh-PDZ revealed that both D319A and L325A mutants reduce GST-Hook interaction, whereas the V322A retained full binding capacity.

We next sought to determine the molecular basis for the Dlg-Hook/Dsh-PDZ interaction. We pursued this question using truncation and/or single amino acid mutants within each interacting domain. The entire Hook domain, including an extended sequence not found in other metazoans (Fig. 2a), was prone to degradation and did not consistently demonstrate robust Dsh binding (Fig. 2b), whereas truncation at proline-749 produced a stable construct capable of Dsh binding. It is also possible that the C-terminal Hook sequence (750–770) induced a conformation that repressed Dsh binding within the N-terminal motif (see below); however, we suspect this would be an artifact of the GST fusion and not a likely contributor to Hook domain function within Dlg itself. We first devised a truncation strategy in which stop codons were inserted incrementally from the Hook C-terminus to produce premature GST-Hook truncations. This approach revealed that truncation up to lysine-712 did not affect the Hook/Dsh interaction; however, truncation at serine-693 completely abolished Dsh binding (Fig. 2a,b). These results indicate that the Dsh-PDZ domain binds an internal peptide ligand located between amino acids 693–712 in the Dlg-Hook domain.

This short sequence within the Hook domain, necessary for Dsh binding, is part of an alternatively spliced segment known as the I3-insert [27], [34], [35]. This region contains prominent sequence homology across multiple species (Fig. 2a), suggesting it plays an important role in Dlg function. The I3-containing Dlg splice isoform is ubiquitously expressed and interacts with 4.1 proteins that aid in Dlg membrane recruitment; whether 4.1 binding affects Dlg structure-function has not been determined, however [35], [36]. The conserved I3-insert sequence contains a polybasic stretch followed by a region of relative hydrophobicity (Fig. 2a). Several strictly conserved residues within this segment were thus selected for alanine mutagenesis analysis. Using Ponceau membrane staining, K700A and F708A showed significant loss of Dsh binding, whereas S705A retained full binding capacity (Fig. 2c, *left*). We also examined binding using western blot analysis against the 6x-His tag on Dsh-PDZ; this more sensitive technique revealed similar results (Fig. 2c, *right*). We conclude that selective, highly conserved amino acids within the Dlg I3-insert mandate a direct interaction with the Dsh-PDZ domain.

We further analyzed the Hook sequence surrounding lysine-700 (699-K-K-N-F-T-F-704), which revealed evident homology to the K-T-x-x-x-W motif within the Fz receptor C-terminus previously identified as a direct Dsh binding sequence [32] (Fig. 2d). Mutation of the lysine, threonine, or tryptophan in this sequence results in reduced Fz/Dsh binding and signaling [32]. Interestingly, Fz sequences typically contain conserved hydrophobic residues C-terminal to this motif, similar to F708 in Dlg. The CXXC zinc-finger protein, Idax, has been shown to bind and inhibit Dsh through a similar motif (K-T-x-x-x-I) [37], and recent studies using phage display-derived peptides optimized for Dsh-PDZ binding revealed a similar sequence homology [38] (Fig. 2d). Among these diverse 6-mer sequences emerges an invariant lysine at the first position along with a conserved hydrophobic residue at the final position, suggesting a K-x-x-x-x-φ sequence may represent a Dsh-PDZ binding motif within diverse binding partners. The second amino acid in this motif is also important (e.g. K700 in Dlg, see Fig. 2c), although it does not appear to be strictly conserved across Dsh-PDZ ligands [32], [37], [38] (Fig. 2d). To explicitly test the requirement of the two conserved positions (referred to as ‘0’ and ‘-5’ based on established PDZ ligand nomenclature [39]), we generated GST-Hook constructs containing either a K699A (-5 position) or F704A (0 position) mutation. Indeed, each of these Dlg mutants were severely impaired in their ability to interact with the Dsh-PDZ domain (Fig. 2e). We conclude that the K-x-x-x-x-φ motif represents a conserved internal Dsh-PDZ ligand sequence found within diverse binding partners. Fz and Idax have a threonine at the second (-4) position and Dlg isoforms have a lysine or arginine (Fig. 2a,d), thus we suggest that K-K/R/T-x-x-x-φ may represent a more generalized internal Dsh-PDZ ligand consensus sequence.

Structural studies of the mouse Dvl2-PDZ domain have found a higher degree of conformational plasticity compared with other PDZ domains, which allows binding of both canonical C-terminal peptides as well as internal ligand sequences to the peptide binding groove formed by the β2-sheet and α2-helix (Fig. 2d) [38]. To determine if Dlg-Hook interacts with the Dsh-PDZ in this ligand binding pocket, we designed a PDZ-L325A mutant, which is a conserved residue located in the α2-helix. This leucine interacts with the terminal hydrophobic amino acid side chain of the K-x-x-x-x-φ motif, as seen in the crystal structure of Dvl2-PDZ bound to two optimized peptide ligands containing either a phenylalanine or isoleucine at the 0 position of the binding motif (Fig. 2d). The PDZ-L325A mutant was impaired in Dlg binding, suggesting that the Hook domain binds within the canonical binding groove of Dsh-PDZ (Fig. 2f). We also targeted a conserved aspartic acid residue in the Dsh-PDZ (D319), as its carboxylate side chain forms a salt bridge with the invariant -5 lysine in K-x-x-x-x-φ. As with L325A, the D319A mutant showed significantly reduced binding to Dlg-Hook (Fig. 2f). Finally, we examined the effects of a V322A mutation on Dlg binding, a residue which also lies along the surface of the α2-helix facing the PDZ binding groove. Despite contacting a tyrosine residue in the N1 and C1 phage peptides [38] (Fig. 2d), no significant effects were seen with respect to Dlg-Hook binding with the L322A mutant (Fig. 2f). Importantly, this position within the ligand consensus motif (the -3 position) is not well conserved among Dsh-binding sequences (Fig. 2d), consistent with its relative importance to PDZ binding. These results collectively demonstrate a biochemical basis for the Dsh-PDZ domain preference for the K-x-x-x-x-φ motif and are consistent with previous findings [32], [37].

### Dsh expression activates Dlg/GukH-mediated spindle orientation

**Figure 3:**
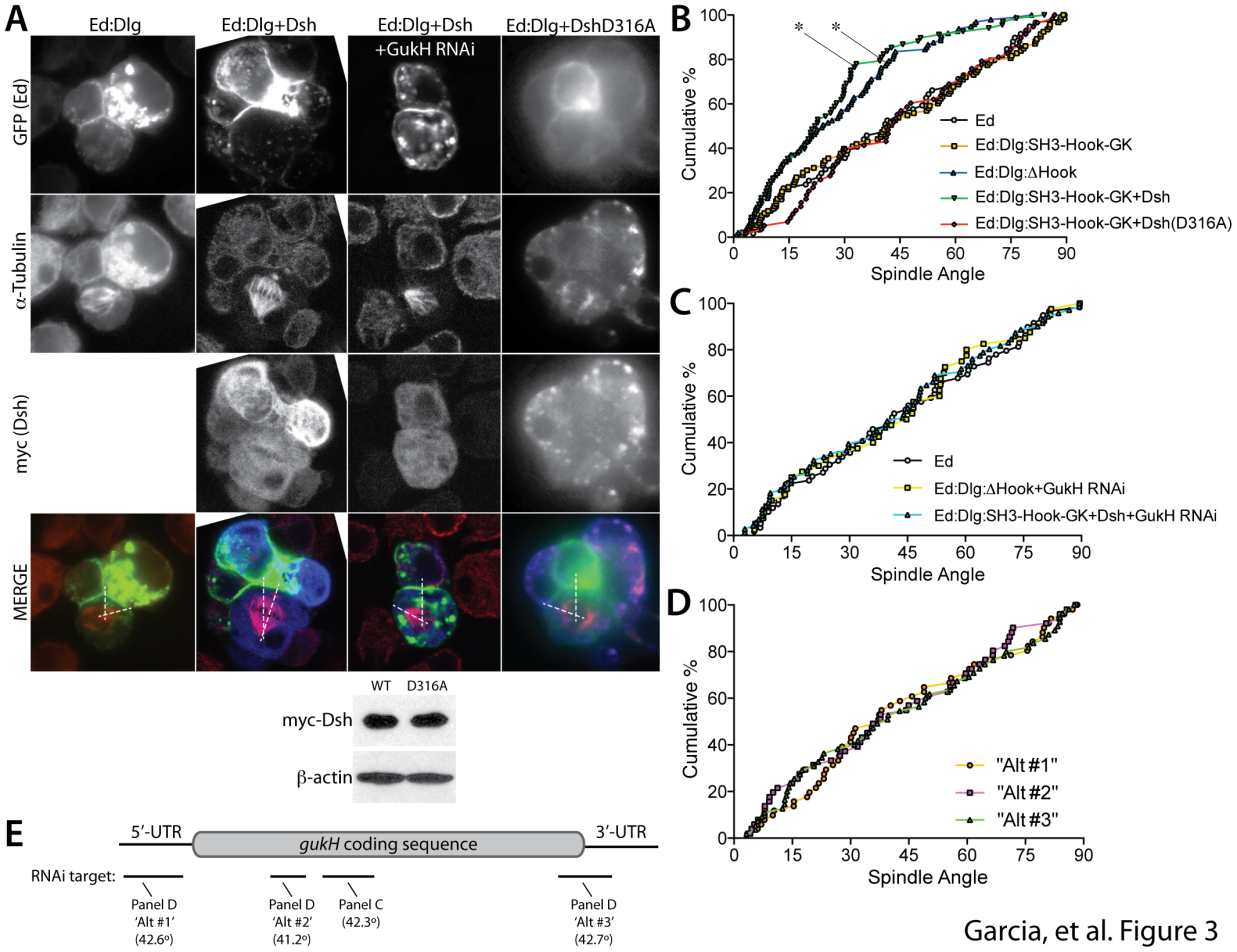
Dsh expression activates Dlg-mediated spindle orientation through the GK interacting protein GukH. (**A**) *Drosophila* S2 cells were transfected with the SH3-Hook-GK domains of Dlg fused to the cell adhesion protein Ed (Ed:Dlg) alone or in combination with full-length myc-tagged Dsh (wild-type or the D316A mutant). In additional experiments, transfected cells were treated with RNAi directed against GukH. ‘Induced polarization’ of cortical Ed:Dlg was achieved through Ed-mediated cell contacts, and spindle orientation angles of mitotic cells were measured relative to the center of the induced Ed:Dlg crescent (*white dashed lines in Merge images*). Shown below cell images are western blots of S2 lysates (20 µg total protein loaded) transfected with either wild-type or D316A Dsh (as pMT:myc fusion constructs), which demonstrate equal protein expression (α-myc). An antibody against β-actin was used as a loading control. (**B**) Cumulative percentage plots reflect fraction of cells with spindles oriented at or below a given angle for all acquired images (n≥30 for all conditions). Ed alone (*black; circles*) and Ed fused to the entire SH3-Hook-GK (*orange; squares*) measurements populate a diagonal expected for completely random orientations between 0-90°. Deletion of the Hook domain (ΔHook; *blue; upward triangles*) or co-expression of Dsh (*green; downward triangles*) results in a leftward shift expected for cell populations with increased percent of lower angle measurements. Co-expression of the non-binding Dsh-D316A mutant (*red; diamonds*) did not improve spindle orientation. *, p <0.05 compared to Ed alone, One-way ANOVA with *Dunnett*'*s post-hoc* test. (**C**) GukH RNAi treatment blocks Dlg-mediated spindle orientation induced by Hook truncation (*yellow; squares*) or Dsh expression (*cyan; upward triangles*) to a level statistically equivalent to Ed alone (*black; circles*). (**D**) Three additional RNAi sequences were generated against distinct sequence elements of GukH and examined for confirmation of specificity. Each alternative (‘Alt #1-3’) target produced a similar loss-of-function phenotype as that in panel C. (**E**) Schematic representation of the GukH transcript and location of each RNAi target (see Methods for primer sequence information). Diverse targets include 5′ and 3′ untranslated regions along with two distinct targets within the coding region. All targets were against sequences shared universally among GukH isoforms. The average spindle orientation angle for each condition is shown in parentheses (compare to 26.3° for Ed:Dlg+Dsh in panel B).

The SH3-Hook-GK cassette of modular domains within Dlg, a hallmark feature of MAGUK family members, is structurally arranged as a supertertiary core that undergoes an intricate interdomain dynamic to ultimately regulate the protein interaction capacity of the GK domain [25], [26], [40], [41]. The GK domain is both necessary and sufficient for Dlg-mediated spindle positioning, and we have previously shown that interdomain dynamics regulate GK output during this process [11], [21], [22], [27], [42]. Specifically, a Dlg construct that includes the entire SH3-Hook-GK is inactive, but deletion of the Hook domain (ΔHook) from this construct is sufficient to induce full GK-mediated spindle orientation activity [27]. We thus wondered whether the Dsh/Hook interaction described above might serve as an intrinsic Dlg activation mechanism within a polarized cellular environment. To address this question, we used our previously established ‘induced polarity’ system in *Drosophila* S2 cells [11]. Here, Dlg constructs are fused to the intracellular C-terminus of the cell adhesion receptor, Echinoid (Ed). Ed-mediated cell adhesion results in cortical redistribution of the Ed:Dlg fusion protein to sites of cell-cell contacts, effectively ‘polarizing’ its localization. Similar to our previous studies, Ed:SH3-Hook-GK was deficient in spindle orientation function, whereas deleting the Hook domain from this protein (Ed:DlgΔHook) significantly improved spindle orientation activity (Fig. 3a,b). Importantly, co-expression of Dsh stimulated spindle orientation activity in the otherwise inactive Ed:SH3-Hook-GK, elevating its function to a level statistically indistinguishable from that of Ed:DlgΔHook (Fig. 3a,b). Dsh expression did not improve random spindle orientation seen with Ed alone (not shown), indicating Dsh effects are specific to Dlg. Furthermore, expression of Dsh-D316A was without effect, despite equivalent protein expression as wild-type Dsh (Fig. 3a), demonstrating that Dsh effects are PDZ-dependent (D316A is the *Drosophila* equivalent to the zebrafish D319A used for *in vitro* binding assays). Together, these results suggest that Dsh binding can modulate the Dlg supertertiary core structure, possibly by disrupting the Hook domain conformation, as an activation mechanism for an important GK-mediated cellular function.

We next sought to understand the molecular basis for Dsh-mediated activation of Dlg. Several Dlg-GK interacting proteins have been identified or implicated as mitotic spindle regulators, including Pins, Khc-73, and Map1a; however, our previous studies suggest these bind Dlg constitutively, bypassing interdomain regulation [21]. In contrast, Dlg binding to GukH is well-established to be under regulatory constraints imposed by SH3-Hook [24], [27]; however, an explicit role for GukH in spindle orientation has not been reported. The SH3-Hook-GK module is deficient in GukH binding, whereas deletion of the Hook domain, either in its entirety or the I3-insert alone, confers robust GukH interaction [27]. These *in vitro* interaction studies perfectly mirror the Hook-mediated regulation of spindle orientation shown above (Fig 3b). Thus, we reasoned that GukH may act downstream of Dlg upon removal of Hook-dependent GK repression. Consistent with this hypothesis, treatment of cells with RNAi directed against GukH completely abolished spindle orientation in cells expressing Ed:DlgΔHook as well as those co-expressing Ed:SH3-Hook-GK and Dsh (Fig. 3c). Three alternative RNAi amplicons were found to have identical effects, supportive of GukH-specific targeting (Fig. 3d,e). We suggest that Dsh binding alleviates Hook-mediated inhibition of a Dlg/GukH-dependent spindle orientation pathway. To our knowledge, these results are the first to identify: (1) a cellular mechanism for the activation of a Dlg-GK-mediated effect, and (2) a mitotic function for GukH. These results also demonstrate the ability of Dlg to orchestrate multiple, diverse downstream binding partners (i.e. Khc-73 and GukH) to regulate a complex biological process.

### Molecular model and implications of the Dsh/Dlg interaction

Crosstalk between Fz/Dsh-mediated Wnt signaling and the Dlg polarity complex has notable implications for animal development. Dlg/GukH plays a key role in the development and organization of the neuromuscular junction (NMJ) in *Drosophila*; loss of Scrib, Dlg, or GukH each result in disorganization and improper function of the NMJ [43]. Interestingly, deletion of the Hook domain results in a loss of cortical Dlg localization at the NMJ synapse [44]. Together with the established role of Wnt signaling in NMJ development [45], these findings suggest the Dsh/Dlg interaction described here may contribute to proper NMJ structure and function. GukH is enriched at the apical cell cortex in *Drosophila* neural stem cells (neuroblasts), where it colocalizes with the Dlg/Scribble/Lgl polarity complex that regulates asymmetric spindle assembly [46]. While it is likely that Dlg requires activation in order to regulate asymmetric neuroblast divisions [42], a direct spindle orientation role for GukH has not been described in these cells, nor has a role for Fz/Dsh signaling been identified.

Planar polarization of *Drosophila* epithelial tissue is controlled by a complex signaling network with numerous regulatory mechanisms [47]. In particular, sensory organ precursor cells (SOPs) rely on these signals for proper asymmetric divisions yielding bristle structures that function within the adult peripheral nervous system. Central to polarity establishment in these cells are the coordinated and mutually-dependent activities of the Fz/Dsh and Pins/Dlg complexes. These codependent functions are necessary for proper cell fate specification that ensures correct bristle development and orientation [9]. Interestingly, these complexes are both known regulators of spindle positioning in SOPs as well, although it should be noted that Dsh and Dlg occupy distinct regions of the cell cortex in dividing SOPs, which complicates a possible role for Dsh/Dlg communication in these cells.

The Fz/Dsh and Dlg polarity complexes are both intimately involved in regulating epithelial cell divisions that lead to development and maintenance of diverse tissue structures. The cortical Dlg complex (Dlg/Scrib/Lgl) is evolutionarily conserved and plays an essential role in apico-basal polarity in diverse cell and tissue types [48]. Maintenance of proper cell polarity serves as a key tumor suppressor mechanism, and mutations in Dlg lead to loss of tissue homeostasis, cellular transformation, and tumorigenesis [49]. Recent studies involving a variety of model cell systems have established a role for the Dlg complex in determining the axis of cell division through regulation of mitotic spindle orientation. Thus, Dlg acts as an important coordinator between cell polarity and cell division orientation, a process that is fundamental to maintaining tissue architecture, promoting proper cell fate specification, and preventing unregulated cell proliferation [11], [15], [50]. Understanding the molecular mechanisms that regulate Dlg function, therefore, has important implications to human health. The dynamic nature of the Dlg ‘supertertiary’ core structure suggest precise regulatory inputs have likely evolved to control its signaling output [25], [26]. Our results demonstrate that Dlg function is regulated through direct interaction with the Wnt signaling effector, Dsh, a protein also well-established as a regulator of cell polarity and proliferation. This regulation is likely to be specific to Dlg splice isoforms containing the I3-insert, the site of Dsh interaction. Previous studies have shown this Dlg segment binds to 4.1 proteins, an interaction that promotes cortical Dlg localization. Whether 4.1 binding alters Dlg function or serves merely as a localization cue remains to be explored [36], [51]. To our knowledge, Dsh represents the first direct Dlg binding partner that activates a GK domain-mediated function within a cellular context.

Although previous studies have focused on the microtubule kinesin motor, Khc-73, in Dlg-mediated spindle orientation, our finding that Dsh/Dlg-mediated spindle orientation is also dependent on GukH highlights a novel component downstream of the cortical Dlg polarity cue. GukH binds the Dlg-GK domain and regulates dynamic localization of the Scribble/Dlg complex at *Drosophila* synapses [43], and is also required for proper neuronal development in the visual system [52]. GukH is the *Drosophila* ortholog of the human Nance-Horan syndrome protein, a developmental disorder characterized by vision impairment, craniofacial abnormalities, and mental retardation [53]-[55]. What role GukH-influenced spindle orientation may play in these processes remains to be explored. Further studies will also be required to delineate the molecular mechanism of GukH activity, although the presence of an N-terminal WASP-homology domain suggests it may play a role in organizing the actin cytoskeleton, which is an important component of spindle assembly and orientation in several systems [56].

Previous studies have linked Dlg to downstream components in the Wnt signaling pathway. Specifically, Dlg binds the Adenomatous Polyposis Coli (APC) tumor suppressor protein [57], an interaction that regulates cell cycle progression [58], cortical polarization, and cell migration [59]. In contrast to the Dsh/Dlg interaction described herein, the APC/Dlg interaction involves a PDZ domain of Dlg binding a canonical C-terminal ligand in APC. The involvement of the APC/Dlg complex in cell polarity likely involves regulation of the cytoskeleton, namely microtubules [60], and may play a role in neuronal development, epithelial morphogenesis, and cancer cell transformation [61]. Our results demonstrate an additional convergence between Wnt/Fz signaling and Dlg, occurring further upstream in the Wnt pathway. Regulation of spindle orientation may contribute to the processes described above, and indeed APC itself has been shown to regulate centrosome and spindle orientation in several model systems [28], [62], [63].

Our results demonstrate a novel interaction between two essential regulators of metazoan development, Dsh and Dlg. This interaction involves a conserved Dlg sequence contained within an alternatively spliced region that shares striking similarity to the Dsh-binding sequence in the Fz receptor. Dsh binding appears to relieve an SH3-Hook domain-mediated inhibition of GK-dependent spindle positioning, which occurs in part through GukH. Further investigation will be required to address the physiological relevance of this model and to what extent a direct Dsh/Dlg complex contributes to spindle positioning *in vivo*.
